# AGGRESCAN: a server for the prediction and evaluation of "hot spots" of aggregation in polypeptides

**DOI:** 10.1186/1471-2105-8-65

**Published:** 2007-02-27

**Authors:** Oscar Conchillo-Solé, Natalia S de Groot, Francesc X Avilés, Josep Vendrell, Xavier Daura, Salvador Ventura

**Affiliations:** 1 Institut de Biotecnologia i de Biomedicina (IBB), Universitat Autònoma de Barcelona, 08193 Bellaterra, Spain; 2 Departament de Bioquímica i Biologia Molecular, Universitat Autònoma de Barcelona, 08193 Bellaterra, Spain; 3 Catalan Institution for Research and Advanced Studies (ICREA), 08010 Barcelona, Spain

## Abstract

**Background:**

Protein aggregation correlates with the development of several debilitating human disorders of growing incidence, such as Alzheimer's and Parkinson's diseases. On the biotechnological side, protein production is often hampered by the accumulation of recombinant proteins into aggregates. Thus, the development of methods to anticipate the aggregation properties of polypeptides is receiving increasing attention. AGGRESCAN is a web-based software for the prediction of aggregation-prone segments in protein sequences, the analysis of the effect of mutations on protein aggregation propensities and the comparison of the aggregation properties of different proteins or protein sets.

**Results:**

AGGRESCAN is based on an aggregation-propensity scale for natural amino acids derived from *in vivo *experiments and on the assumption that short and specific sequence stretches modulate protein aggregation. The algorithm is shown to identify a series of protein fragments involved in the aggregation of disease-related proteins and to predict the effect of genetic mutations on their deposition propensities. It also provides new insights into the differential aggregation properties displayed by globular proteins, natively unfolded polypeptides, amyloidogenic proteins and proteins found in bacterial inclusion bodies.

**Conclusion:**

By identifying aggregation-prone segments in proteins, AGGRESCAN  shall facilitate (*i*) the identification of possible therapeutic targets for anti-depositional strategies in conformational diseases and (*ii*) the anticipation of aggregation phenomena during storage or recombinant production of bioactive polypeptides or polypeptide sets.

## Background

Protein aggregation has become a key topic in both biotechnological and medical sciences [1,2]. It constitutes the main bottleneck in protein production, narrowing the spectrum of relevant polypeptides obtained by recombinant techniques [3]; it reduces the shelf life and increases the immunogenicity of polypeptidic drugs [4]; and it is associated with an increasing number of critical human diseases including Alzheimer's disease, spongiform encephalopaties, type II diabetes mellitus and Parkinson's disease [5-8].

In the last decade data have begun to accumulate suggesting that the composition and the primary structure of a polypeptide determine to a large extent its propensity to aggregate and that small changes may have a huge impact on solubility. The ability to predict the aggregation propensity of a protein from its sequence would be of much value, for example, in the control of unwanted protein deposition events through specific sequence targeted therapeutics or in the discovery of more soluble variants of proteins of biotechnological interest. It is commonly assumed that not all regions of a polypeptide are equally important in determining its aggregation tendency. In this context, some authors have recently proved that very short specific amino acid stretches can act as facilitators or inhibitors of amyloid fibril formation [9,10]. These relevant regions are usually known as aggregation "hot spots" (HS) and their presence has been described in most of the peptides and proteins underlying neurodegenerative and systemic amyloidogenic disorders [11].

In previous work we exploited the experimental data obtained from a system *in vivo *that uses the β-amyloid peptide as model to derive a simple approach for the detection of "hot spots" of aggregation [12,13]. This approach permitted the identification of aggregation-prone segments in several unstructured and globular disease-linked polypeptides and the prediction of the effect of disease-linked mutations in some of these polypeptides. Here, we describe a software and web interface (AGGRESCAN) that implement this approach and extend it to the general prediction of aggregation "hot spots" and the evaluation of their contribution to the differential aggregation behaviour of polypeptides. In addition to enabling the simultaneous analysis of a large number of sequences, AGGRESCAN introduces a new set of functions and descriptors for the identification of "hot spots" of aggregation and the determination of their relevance within the parent sequence.

## Implementation

### Approach

Recent findings in the study of protein aggregation indicate that not all the polypeptides share the same aggregation propensities and that there exists specific continuous protein segments that can nucleate the aggregation process when exposed to solvent [9,10], suggesting a sequence-dependence of aggregation propensities. At the same time, it has been shown that the same physicochemical principles underlie the aggregation propensities of different polypeptides from unfolded states [14]. According to these assumptions one may expect that the conclusions obtained from the study of a relevant nucleating sequence, or "hot spot" of aggregation, in its natural polypeptidic context could apply to other unrelated proteins. Using an in vivo reporter method to study a "hot spot" in the central hydrophobic core of Aβ we calculated the effect of single point mutations on the aggregation propensities of the peptide within the cell. The results were used to approximate the *in vivo *intrinsic aggregation propensities of natural amino acids when located in an aggregation-prone sequence stretch [12] (see additional file 1). This information was subsequently used to generate an aggregation profile for any protein sequence under study to detect those regions with high aggregation propensities. Comparison of the theoretically calculated changes in aggregation propensities between a wild type sequence and different mutants serves also as a tool to predict the behavior of the mutant forms. Albeit the basic simplicity of this phenomenological model, it predicts, at least qualitatively, both the presence of experimentally validated "hot spots" and the variations in aggregation propensity introduced by mutations in some disease-related polypeptides [13].

### System description

AGGRESCAN is a web-based tool with a computing core coded in C and a front end written in a combination of html and perl cgi. Development of AGGRESCAN was carried out under Mandriva Linux LE2005 and the service is currently running under Mandrake Linux 9.0 on a Pentium 4 1300 MHz (willamette) with 1GB RDRAM.

For each polypeptide sequence input, AGGRESCAN calculates and reports: *i*) an aggregation-propensity value for each residue in the sequence and a graphical representation of the profile for the entire polypeptide; *ii*) the areas of profile peaks over a precalculated threshold and a graphical representation of peak-area values; *iii*) putative aggregation "hot spots", identified from the polypeptide's aggregation profile.

#### Input

The polypeptide sequence(s) can be typed or pasted on screen using FASTA format. Despite supporting up to 100 characters for name entries, use of very long names is discouraged as it disturbs the visualization of the output. Sequence entries may not contain more than 2,000 residues and the letters must correspond to those associated to the 20 natural amino acids. If these two conditions are not satisfied an error message will appear on screen. White-space, enter and tab characters are ignored. Characters may be entered as lower and/or upper case, and so will remain in the output.

#### Processing

The calculations are based on aggregation-propensity values per amino acid (aaAV, or a3v) derived previously from experimental data [12]. The program calculates the a3v average (a4v) over a sliding window of a given length and assigns it to the central residue in the window. The size of the sliding window ([5,7,9], and [11] residues) was trained against a database of 57 amyloidogenic proteins, in which the location of "hot spots" was experimentally known. In general, the predictions of the overall aggregation-prone regions do not depend on the length of the used windows and only slightly affect their limits. There are, however, two remarkable exceptions: 1) The use of long windows on top of very short sequences results on excessive smoothing of the profile and experimentally different "hot spots" become grouped and masked and cannot be individualized in the prediction. 2) The use of short windows on top of very long sequences results in the appearance of a number of short experimentally non-relevant predicted "hot spots" with associated low areas. Thus, the procedure incorporates a ponderation of the window length relative to the size of the analyzed protein. The best predictions were obtained using a window size of 5 for < = 75 residues, 7 for < = 175, 9 for < = 300 and 11 for > 300, respectively, probably reflecting that for longer sequences larger "hot spots" are necessary in order to significantly increase their aggregation propensities, while short-stretches suffice for smaller peptides. To account for charge effects at the polypeptide's termini (NH_3 _^+ ^and COO^-^) a virtual residue is added to each side of the chain (residue 0 at the N-terminus and residue n+1 at the C-terminus, n being the original sequence length). The a^3^v of residue 0 equals the average a^3^v of the basic residues (K, R), while that of residue n+1 equals the average a^3^v of the acidic residues (D, E). The first window, ranging from residue 0 to residue 4, 6, 8 or 10 (depending on window size), will serve to assign an a^4^v to residue 2, 3, 4 or 5, respectively. Thus, the off-centre residues 1, 1–2, 1–3 or 1–4 may not have an associated a^4^v. This is solved by giving these residues the value corresponding to the first window centre. The same procedure is followed at the C-terminus. The "hot spot" threshold (HST) has been defined as the average of the a^3^v of the 20 amino acids weighted by their frequencies in the SwissProt database [15]. The aggregation profile (AP) of the polypeptide is defined by the complete sequence of a^4^v. The sum of a^4^v and the average of a^3^v over the entire sequence (a^4^vSS and a^3^vSA, respectively) are also calculated. A region in the polypeptide sequence is considered an aggregation "hot spot" (HS) if there are 5 or more sequentially continuous residues with an a^4^v larger than the HST and none of them is a proline (aggregation breaker) [16]. The average a^4^v in each "hot spot" is then calculated (a^4^vAHS). Finally, the area of the AP above the HST (AAT), the total area (TA, HST being the zero axis), and the area above the HST of each profile peak identified as "hot spot" (HSA) are integrated numerically using the trapezoidal rule (see additional file 2).

#### Output

With current service resources, the delay time between pressing the submit button and receiving the output on screen is of 10 minutes for an input set of 100 sequences of sizes between 40 and 1,000 residues. The output is structured in tables, one per sequence and an additional one with averages over all sequences, an excel-readable document with output values and a list of sequences sorted by normalized a^4^vSS for 100 residues (Na^4^vSS). The first row in the output contains the sequence names. The second row displays links to the three graphics produced per sequence, i.e., Profile graphic: AP (red), a^3^vSA (green), HST (blue); Area graphic: HSA (same value assigned to all residues in the "hot spot"); Normalized-Area graphic: normalized HSA for a 100-residue "hot spot" (NHSA). In the following rows we find the a^3^vSA, the number of "hot spots" identified (nHS), the normalized number of "hot spots" for 100 residues (NnHS), the AAT, the THSA, the TA, the AAT and THSA divided by the number of residues (AATr and THSAr, respectively), and Na^4^vSS. Finally, a row per residue is given with columns for the residue number, its one-letter code, a^4^v, HSA, NHSA, and a^4^vAHS (see additional file 3).

## Results and Discussion

### AGGRESCAN capabilities: Validation and Examples

#### Generation of protein aggregation profiles and prediction of aggregation "hot spots"

The prediction method implemented in AGGRESCAN has already allowed the identification of experimentally proved "hot spots" (HSs) in a set of both natively unfolded and globular pathogenic proteins: Aβ42 peptide, synuclein, amylin, prion protein, transthyretin, β2-microglobulin and lysozyme [12]. The main aims in the design of AGGRESCAN were the automation of this analysis for the study of large sets of polypeptide sequences, the introduction of new variables in the postprocessing of the aggregation profiles to provide a set of values that could be easily correlated with aggregation propensities and the presentation of results in a convenient and informative way. To further prove the general predictive ability of the method, the above-mentioned proteins, together with a new set of well studied protein sequences related to depositional diseases (aDan, aBri, apolipoproteins AI, AII, AIV, and CII, prolactin, insulin, Tau, fibrinogen, amyloid A, pulmonary surfactant protein, tropoelastin and medin), or shown to form amyloid *in vitro *(myoglobin, glycophorin A and amphoterin) have been analyzed with AGGRESCAN (Table 1). The predicted aggregation-prone protein regions have been validated by comparison to available experimental data on (i) regions known to promote aggregation, (ii) fragments known to aggregate *in vivo *(often after proteolysis) and (iii) synthetic short peptides shown to aggregate *in vitro *(references in Table 1). In the AGGRESCAN output, the sequence stretches with highest predicted aggregation propensity are shown in red in the peptide sequence column and appear as peaks in the Profile plots. The HS can be ranked according to their peak area (HSA) or normalized peak area (NHSA). Interestingly, protein segments that are experimentally known to be involved in aggregation are also found among the top ranked HS in their respective sequences based of the approach described here (Table 1), indicating that AGGRESCAN catches the main features underlying deposition in many conformational diseases. These results, together with previous experimental [10,17-20] and theoretical [21-24] data, suggest that specific short polypeptide stretches effectively promote and/or modulate protein amyloid formation.

**Table 1:** List and ranking of the predicted aggregation-prone regions in the different disease-linked polypeptides analyzed in this study and comparison with the available experimental data.

**Protein**	**Experimental region**^**a**^	**Predicted region**^**b**^	**Ranking**^**c**^	**References**
**Abri**	1–34	4–9	2/2	[52]
		15–28	1/2	
**Adan**	1–34	4–9	2/2	[53]
		15–24	1/2	
	68–78	66–77	1/6	[54–56]
**α-Synuclein**	31–109	36–42	2/6	[56]
		49–55	4/6	
		87–94	5/6	
**Amphoterin**	12–27	14–22	2/3	[57]
**Amyloid-β-protein**	17–21	17–22	2/2	[58]
	31–36/38–42	30–42	1/2	
**Apoliprotein A-I**	1–83	13–21	2/2	[59]
**Apoliprotein A-II**	N-terminal fragments	1–19	1/3	[11]
**Apoliprotein A-IV**	N-terminal fragments	1–19	1/6	[11]
**Apoliprotein C-II**	57–74	60–67	2/3	[60]
		69–76	1/3	
**β2-Microgobulin**	21–41	22–30	2/2	[61]
	59–79	59–70	1/2	[62]
**Exon 30 Tropoelastin**	1–25	1–7	2/2	[63]
		9/18	1/2	
**Fibrinogen A α-chain**	501–506	499–521	1/6	[64]
	482–504	501–506	1/5	
**Glycophorin A**	70–98	74–98	1/4	[65]
**Insulin**	1–38	12–19	1/3	[66]
		21–27	3/3	
**Islet amyloid polypeptide**	8–20	13–18	1/2	[67]
	20–29	24–28	2/2	[68]
**Lysozyme (Hen)**	40–64	54–62	2/4	[69]
	49–101	76–84	3/4	[70]
**Medin**	47–54	49–55	1/3	[71]
**Myoglobin (Horse)**	101–118	101–115	1/4	[72]
**Prion Protein**	106–147	117–136	3/6	[73]
		138–142	6/6	
**Prolactin**	1–34	10–32	2/9	[74]
**Pulmonary surfactant protein**	24–58	31–59	1/5	[25]
**Serum Amyloid A**	2–12	1–9	1/2	[75]
**Tau**	301–320	304–311	1/2	[27]
	10–20	12–19	2/7	[76]
**Transthyretin**	105–115	105–112	3/7	[77]
		114–123	4/7	

^a^Sequence stretches experimentally identified as critical for protein aggregation.

^b^Coincident aggregation-prone segments as predicted by AGGRESCAN.

^c^The rank position refers to the entire protein and reflects the importance of this specific "hot spot" (HS) relative to all the aggregation-prone regions identified by AGGRESCAN in the protein. (i.e., 1/4 indicates that this HS has the highest aggregation propensity of the four detected in a particular sequence by the software)

**Figure 1:**
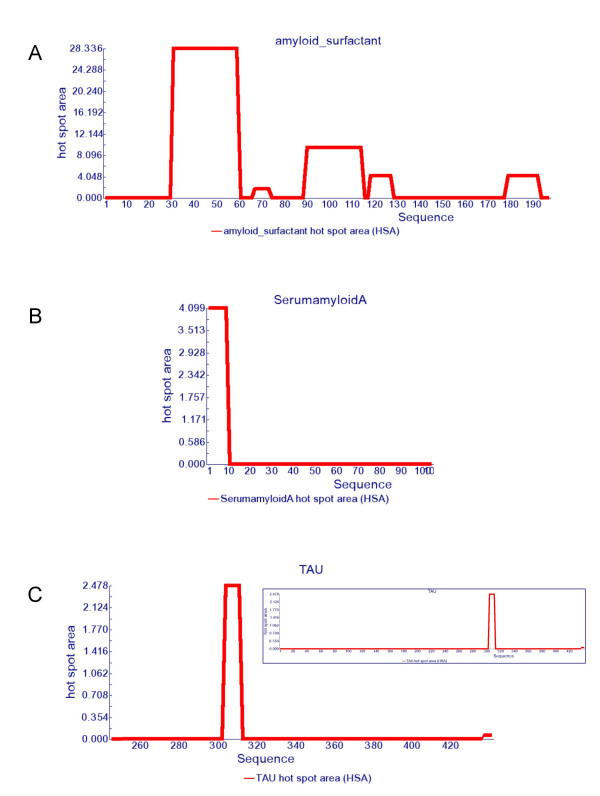
**Hot spot area graphics**. Hot spot area plots for a) lung surfactant protein C, b) serum amyloid A protein and c) Tau protein.

One remarkable example in the test set is lung surfactant protein C (SP-C). This protein is expressed as a 197-amino acid proprotein that is processed to the 35-amino acid mature peptide. This fragment is associated with the development of pulmonary alveolar proteinosis (PAP). The bronchoalveolar fluid from PAP patients is rich in insoluble SP-C aggregates which exhibit the characteristic properties of amyloids by Congo red staining and electron microscopy. Moreover, the isolated peptide has been shown to form amyloid fibrils *in vitro *[25]. In good agreement with this data, AGGRESCAN predicts the SP-C region within the precursor as the HS with the highest aggregation propensity (Figure 1).

Other two interesting molecules are serum amyloid A (SAA) and Tau proteins, involved in systemic amyloidosis and Alzheimer's disease, respectively. AGGRESCAN detects only one HS in SAA and a very dominant one in Tau (Figure 1). In both cases, these sequence stretches correspond to the unique regions in SAA and Tau proved to be relevant for amyloidosis [26,27]. Importantly, the SAA and Tau sequences display highly negative Na^4^vSS values, -28.2 and -32.5 respectively. Although this suggests an overall low aggregation propensity, the presence of specific HS that can act as nucleation points from which the ordered fibrillar structure can be expanded under certain circumstances, turn these proteins amyloidogenic. Actually, Tau is an usually highly soluble microtubule-associated protein [28] but in Alzheimer's disease it aggregates into fibres with a tendency to form neurofibrillary tangles.

To date, only few 3D structures of amyloid assemblies at atomic resolution are available [29]. A crucial question is whether the formation of the tightly packed β-sheets observed in these structures is a generic backbone property or is dictated by the sequence. Interestingly enough, AGGRESCAN detects the presence of "hot spots" in most of the strands forming the intimate structure of the different protein fibrils (Table 2), providing additional support for the relevance of the primary structure on amyloid formation.

**Table 2:** Comparison of AGGRESCAN predictions with the structural composition of different amyloid fibrils.

**Protein**	**Structure (β-strands)**	**Prediction**	**Reference**
**Aβ1-40**	β 1: 12–24	17–22	[78]
	β 2: 30–40	30–40	
**Amylin**	β 1: 12–17	13–18	[79]
	β 2: 22–27	24–28	
	β 3: 31–37	-	[80]
**HET's Prion**	β 1: 226–234	-	
	β 2: 237–245	238–248	
	β 3: 262–270	263–267	
	β 4: 273–282	272–276	
**Mouse Prion (89–143)**	β 1: 112–124	115–129	[81]
**β2- microglobulin (20–41)**	β 1: 21–30	22–30	[82]
	β 2: 33–40	-	
**Transthyretin (105–115)**	β 1: 105–115	105–112	[83]

**Figure 2:**
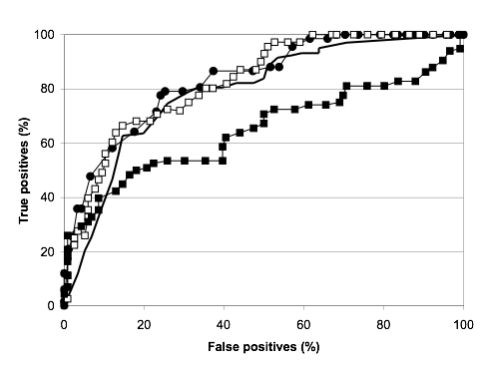
**Comparative prediction performance of AGGRESCAN and structure-based methods**. Comparative predictions of AGGRESCAN (solid circles), packing density profile [31] (no symbols), 3D Profile [32] using the NNQQNY template (solid squares) and 3D Profile using an ensemble of templates (empty squares). Predictions were tested in a Database of Fibril Formers and Non-Formers hexa-peptides. Predictions are shown as receiver-operator characteristic curves.

There are several computational approaches for detecting aggregation-prone regions and predicting polypeptide propensities for amyloid fibril formation. Some of them, including AGGRESCAN, rely on experimental or theoretical calculations of individual amino acid aggregation propensities and on the use of these values to scan protein sequences. The main difference between these algorithms is the way aggregation propensities are obtained. Pawar and co-workers proposed an aggregation scale based on phenomenological expressions relating protein intrinsic factors with the aggregation rates of a set point mutants scattered along acylphosphatase sequence and of a few other polypeptides [30]. As the fitting was done considering effects in both aggregation relevant and non-relevant regions, it is possible that the data do not necessarily reflect propensities within nucleating sequences. To address this point, Rojas Quijano and co-workers derived propensities from the analysis of the Tau-related amyloidogenic peptide Ac-VQIVYK-amide and its single site mutants Ac-VQIVXK-amide (X≠Cys) [19]. In AGGRESCAN, we somehow combine both approaches, in the sense that (i) propensities are calculated from the analysis of single mutants in a nucleating sequence (the central hydrophobic cluster of Aβ) which is perhaps the best well characterized aggregation-prone sequence in the literature and one of the few for which a high-resolution structure in the amyloid conformation is available, and (ii) we consider it in the context of the full length polypeptide (in fact fused to GFP, which acts as aggregation reporter) and not in an isolated manner as a short peptide. In addition, to the best of our knowledge our method is the only one in which aggregation propensities have been derived from experiments inside the cell, where the presence of the folding machinery might modulate the aggregation tendencies of polypeptides. Besides these three experimentally calculated propensity scales, Galzitskaya and co-workers have used the mean packing density for natural amino acid residues in protein structures, as a scale to predict amyloidogenic regions in proteins [31]. A comparative analysis of the four different scales shows that, despite these differences, there is a striking correlation between our in vivo obtained amino acid aggregation propensities and the others (Table 3), probably because they reflect a combination of properties characteristic of protein aggregation, such as hydrophobicity, secondary structure propensity or packing density. Importantly, although our method was not aimed at the specific identification of short amyloidogenic peptides, but rather of aggregation-prone sequences within natural proteins, AGGRESCAN identifies the presence of at least one hot spot in more than 80% of the amyloid forming sequences in a set of experimentally characterized peptide fragments of amyloidogenic proteins [32]. Also, using a database of six-residue peptides containing both amyloid formers and non-formers [32,33] the receiver operator characteristic (ROC) curve for our method compares well with those obtained using structure-based data, such us packing density on protein structures or the 3D profile method, based on the threading of six-residue peptides through the known crystal structure of the cross-β spine formed by the peptide NNQQNY from Sup35 yeast prion [32] (Figure 2).

**Table 3:** Correlation coefficients (R) between the individual amino acid aggregation propensities used by AGGRESCAN and those used by other predictive methods.

	AGGRESCAN	AMYLOID1^a^	AMILOYD2^b^	AMYLOID3^c^
AGGRESCAN	*	0.849	0.794	0.867
AMYLOID1^a^	0.849	*	0.764	0.837
AMILOYD2^b^	0.794	0.764	*	0.807
AMYLOID3^c^	0.867	0.837	0.807	*

^a ^AMYLOID1 corresponds to the method described in Ref. [22]

^b^AMYLOID2 corresponds to the method described in Ref. [19]

^c^AMYLOID3 corresponds to the method described in Ref. [31]

Overall, the success of different computational approaches in predicting aggregation-prone regions allows to propose that aggregation propensity in polypeptide chains is ultimately dictated by the sequence. As it happens with the native conformation of proteins, the sequence contains intrinsic information that is relevant for the regular structural arrangement within β-aggregates, implying that the mechanism of amyloid fibril formation is similar for different peptides and proteins.

#### Prediction of the effects of protein mutation on the aggregation propensity

Aggregation propensity varies sensibly with the composition and especially the sequence of the polypeptide, in such a way that single amino acid substitutions in proteins associated to depositional diseases result in many cases in changes in the global protein aggregation propensity and sometimes lead to premature or acute pathological symptoms. Predicting the effect of a mutation on the aggregation tendency of a protein could help to anticipate the implications of that mutation in disease development or assist the design, production and storage of more soluble variants of biotechnologically relevant proteins and peptides [34].

Several AGGRESCAN output variables can be used to predict the effect of sequence variations on the aggregation propensities of a given polypeptide. The change in the normalized a^4^v sum (Na4vSS) and Total Area (TA) are obvious indicators of changes in aggregation properties of the complete sequence due to point mutations. Nevertheless, a mutation will not always affect significantly the global profile and changes in the number of HS (nHS), in the area over the HS threshold (AAT) or in the area assigned to the HS regions (THSA), are also informative. The normalized values (relative to the number of residues) AATr, THSAr and NHSA should be used if mutations resulting in sequence deletions or insertions are considered. To asses the capability of AGGRESCAN to predict sequence-variation effects we compared the experimentally observed aggregation changes reported in the literature for a group of more than 50 protein mutations with the change in different AGGRESCAN output variables. The analysis indicates that Na^4^vSS is a good predictor for the effect on aggregation propensity changes in the polypeptide sequence on aggregation propensity (Table 4). The user has to take into account that a given mutation in a short protein is expected to have higher impact on aggregation that the same change in a longer sequence, where its effect can be more easily modulate by the rest of the sequence. These considerations are already included in the calculation of the Na4vSS values.

**Table 4:** Comparison of the predicted and experimentally tested effects of mutations on the aggregation propensity of amyloidogenic proteins.

**Sequence Name**	**ΔNa**^**4**^**vSS**^**a**^	**Experimental**^**b**^	**References**
**Peptide Aβ42 A21G**	-16	-	[84]
**Peptide Aβ42 E22K**	15	+	[84]
**Peptide Aβ42 E22G**	29	+	[84]
**Peptide Aβ42 E22Q**	5	+	[84]
**Peptide Aβ42 F19P**	-68	-	[85]
**Peptide Aβ42 F19T**	-63	-	[35]
**Peptide Aβ42 D23N**	16	+	[86]
**Peptide Aβ42 F19D**	-118	-	[12]
**Peptide Aβ42 I31L**	-15	-	[87]
**Peptide Aβ42 I32L**	-15	-	[87]
**Peptide Aβ42 I41G**	-62	-	[87]
**Peptide Aβ42 I41A**	-49	-	[87]
**Peptide Aβ42 I41L**	-12	-	[87]
**Peptide Aβ42 A42G**	-10	-	[87]
**Peptide Aβ42 A42V**	32	+	[87]
**Peptide Aβ42 Δ 1–4**	59	+	[88]
**Peptide Aβ42 Δ 1–9**	237	+	[88]
**Peptide Aβ42 Δ 40–42**	-63	-	[88]
**Peptide AβgΔ 41–42**	-34	-	[36]
**Peptide Aβg5**	89	+	[36]
**Peptide Aβg6**	111	+	[36]
**Peptide Aβg7**	167	+	[36]
**Peptide Aβ42 V12E+V18E+M35T+I41N**	-312	-	[87]
**Peptide Aβ42 F19S+L34P**	-123	-	[87]
**TAU R5L**	2	+	[89]
**TAU R406W**	2	+	[90]
**TAU G272V**	2	+	[90]
**TAU Y310W**	0	=	[39]
**TAU P301L**	1	+	[40]
**TAU S320F**	2	+	[91]
**α-synucleinA30P**	-1	=	[92]
**α-synucleinE46K**	2	+	[92]
**α-synucleinA53T**	-1	+	[92]
**α-synucleinA76E**	-5	-	[93]
**α-synucleinA76R**	-3	-	[93]
**Amylin (Rat) R18H**	9	+	[94]
**Amylin (Rat) L23F**	17	+	[94]
**Amylin (Rat) V26I**	11	+	[94]
**Amylin (Rat) R18H+L23F+V26I**	34	+	[94]
**Amylin (human) (22–27) N22A**	21	+	[68]
**Amylin (human) (22–27) F23A**	-59	-	[68]
**Amylin (human) (22–27)G24A**	16	+	[68]
**Amylin (human) (22–27) I26A**	-61	-	[68]
**Amylin (human) (22–27) L27A**	-23	-	[68]
**Amylin (human) S20G**	-106	+	[95]
**Amylin (human) ProIAPP**	-90	+?	[96]
**Human PrP H111A**	5	+/=	[97]
**Human PrP H111K**	0	-/=	[97]
**Human PrP A117V**	7	+	[97]
**Human PrP V210I**	1	+	[98]
**Stefin R68X**	37	+	[41]
**Stefin G4R**	-6	-	[41]
**SH3 n47a**	17	+	[46]

^a^Relative change in Na^4^vSS upon mutation, expressed as percentage.

**ΔNa**^**4**^**vSS = ((Na**^**4**^**vSS**_**mut**_** - Na**^**4**^**vSS**_**wt**_**)/|Na**^**4**^**vSS**_**wt**_|)*100

**Na**^**4**^**vSS**_**mut **_refers to the Na^4^vSS value of the mutant sequence.

**Na**^**4**^**vSS**_**wt **_refers to the Na^4^vSS value of the wild type sequence.

^b^Changes in aggregation determined experimentally.

Symbols: + increase; - decrease; = no significant change.

**Figure 3:**
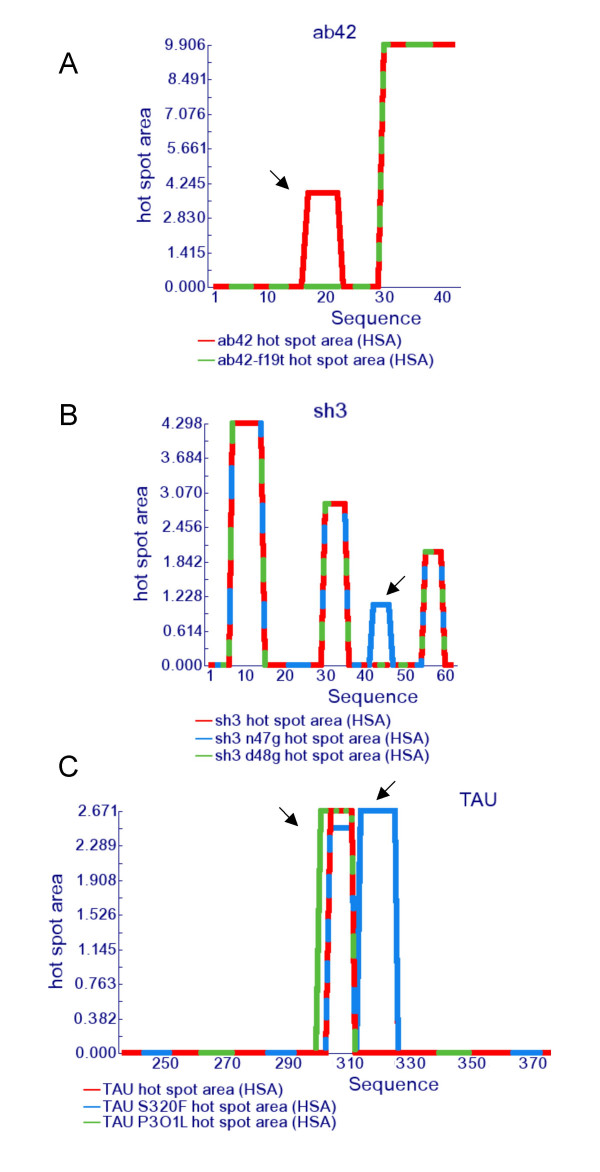
**Changes in the hot spot area plot caused by point mutations in amyloidogenic proteins**. a) Aβ42 wild type (red) and Aβ42 F19T mutant (green). b) SH3 wild type (red), SH3 D48G (green) and SH3 N47G (blue). c) TAU wild type (red), TAU P301L (green) and TAU S320F (blue).

The algorithm predicts accurately a large set of natural and designed mutations of Aβ42 (the central hydrophobic region of this peptide was used for the derivation of the current a^3^v parameter set of AGGRESCAN) (Table 4). As an example, Figure 3 shows how the F19T mutation, which strongly decreases the deposition of Aβ42 [35], results in the loss of the central HS in this peptide. Interestingly, it also anticipates the lower aggregation propensity of Aβ40 and the recent observation that longer Aβisoforms possess increased aggregation propensities [36]. Several natural occurring mutations have also been shown to affect the aggregation rate of Tau [37-40]. The predicted changes in the respective Na^4^vSS correlate well with the experimental changes observed in these Tau variants (Table 4). Figure 3 shows the Area plot of wild type Tau and two of its mutants with highest, experimentally tested, aggregation propensities. The P301L substitution increases by 1,4 fold the area associated to the main "hot spot" in Tau. In addition, AGGRESCAN predicts the presence of a new HS in the S320F mutant, absent in the wild type form. This mutant is linked to tauthopaty, in which Tau accumulates in inclusion bodies [40].

Other disease-related protein mutants studied here are the recently described G4R and R68Stop of human Stefin B protein. These mutants have been related with the development of Myoclonus epilepsy of type 1. It has been described that R68Stop is more prone to aggregate than wild type Stefin, while the G4R mutant shows an opposite behavior, with a slower fibril formation rate [41]. In agreement with these experimental observations the algorithm predicts an increase in the Na^4^vSS associated to the R68Stop mutation and a decrease for the Gly4Arg change (Table 4).

Type 1 serum amyloid A protein (SAA1) is associated with Familial Mediterranean fever (FMF). FMF patients' genotypes are thought to correlate with the different phenotypes of the disease. A recent study [42] concludes that the gamma SAA1 allele is more frequently observed in the population devoid of amyloidosis, thus suggesting a protective effect of this allele on the development of the illness. In agreement with these results the AGGRESCAN analysis of amyloid A sequence variants predicts that the gamma variant misses a HS and has a lower Na^4^vSS than other alleles.

The Src homology 3 (SH3) domain of the p58 subunit of phosphatidyl-inositol-3 -kinase (PI3-SH3) is one of the best-characterized examples of a small globular protein unrelated to any known pathological condition that can form amyloid fibrils *in vitro *[43]. Aggregated species obtained from this protein have been found to be cytotoxic when added to cell cultures [44]. We have previously shown that the α-spectrin-SH3 (SPC-SH3) domain, which shares the same fold and 24% sequence identity with PI3-SH3, is a soluble protein that does not form amyloid fibrils under any conditions tested [45]. Nevertheless, a recent work found that the N47A mutation at the distal loop induces the formation of amyloid fibrils [46]. In contrast, the mutation of residue 47 to Gly does not promote aggregation (Ventura, S., unpublished results). According to AGGRESCAN a new HS occurs in the amyloidogenic mutant relative to both the wild type and N47G species, which could be responsible for its increased aggregation abilities (Figure 3).

#### Analysis of protein datasets

Besides analyzing the theoretical aggregation properties of single molecules and their individual mutants, AGGRESCAN is also able to deal simultaneously with a large number of sequences. This ability can be specially useful to compare the global aggregation properties of different protein sets and may help to delineate general rules underlying the relationship between the primary structure of proteins and peptides and their specific *in vivo *and *in vitro *depositional properties. With this aim we studied the correlation between the structural/aggregational features of 5 different groups of proteins and the predictions provided by AGGRESCAN. These datasets were: 1) natively globular proteins (160 proteins) (from SCOP, the ASTRAL40 set); 2) natively intrinsically unstructured proteins (51 proteins); 3) proteins which are soluble when overexpressed in bacteria (38 proteins); 4) proteins forming inclusion bodies when overexpressed in bacteria (121 proteins) and 5) amyloidogenic proteins (57 proteins) (see additional file 4).

When average AGGRESCAN output values are calculated and subsequently compared between data sets, it appears that the different protein groups can be individualized (Table 5), providing insight into the sequential determinants of protein aggregation and solubility. In this way, intrinsically unstructured proteins (IUP) clearly present the lowest output values of all datasets, in correlation with the general observation that unstructured proteins are usually resistant to aggregation and remain soluble after heat-treatment of the cells. Natively unfolded proteins exhibit a Na^4^vSS value 7 times lower than that corresponding to the set of globular proteins from SCOP. Also, the normalized number of HS (NnHS) or the area over the threshold (AAT) and total HS area (THSA) are around 2 times higher in globular proteins than in IUP, showing that, in agreement with other automated analyses [47], the number of aggregation-prone sequence stretches is lower in IUP than in structured proteins. This result may reflect a negative natural selection against aggregation promoting residues and regions in IUP, where any HS will be exposed to solvent and accessible for the establishment of inter-molecular contacts that may finally lead to the build-up of aggregates. For the same reason, nature is likely to have provided globular proteins with a stable native conformation in which aggregation-prone sequences are buried in the inner hydrophobic core or involved in intra-molecular interactions [13,18]. This appears to be a successful evolutive strategy to avoid deposition, since few proteins aggregate from their folded state. Hence, amyloidogenic mutations in globular proteins usually result in destabilization of the native state, permitting exposure of natively hidden HS.

**Table 5:** Comparison of the different AGGRESCAN parameters for globular, natively unstructured, amyloidogenic, soluble and insoluble proteins.

**Set Name**	**Globular**^**1**^	**Unfolded**^**2**^	**Amyloid**^**3**^	**IBs**^**4**^	**Soluble**^**5**^
***a3vSA***	***-0.04***	***-0.28***	***-0.12***	***-0.02***	***-0.05***
**nHS**	9.54	5.63	5.86	11.97	10.34
***NnHS***	***3.89***	***2.06***	***2.89***	***3.50***	***3.35***
**AAT**	29.94	18.21	24.51	41.27	34.43
**THSA**	25.58	14.97	21.26	36.00	29.61
**TA**	-5.17	-60.95	-26.42	-5.00	-5.55
***AATr***	***0.12***	***0.07***	***0.13***	***0.13***	***0.12***
***THSAr***	***0.11***	***0.05***	***0.11***	***0.11***	***0.09***
***Na4vSS***	***-4.26***	***-28.73***	***-12.96***	***-2.51***	***-5.18***

In bold and italics are shown those parameters that are normalized by the number of residues, allowing direct comparison of datasets independently of protein size.

^1^Natively globular proteins: 160 proteins randomly selected from SCOP (the ASTRAL40 set)

^2^Natively intrinsically unstructured proteins: 51 proteins

^3^Amyloidogenic proteins: 57 proteins

^4^Proteins forming inclusion bodies when overexpressed in bacteria: 121 proteins

^5^Proteins which are soluble when overexpressed in bacteria: 38 proteins

**Figure 4:**
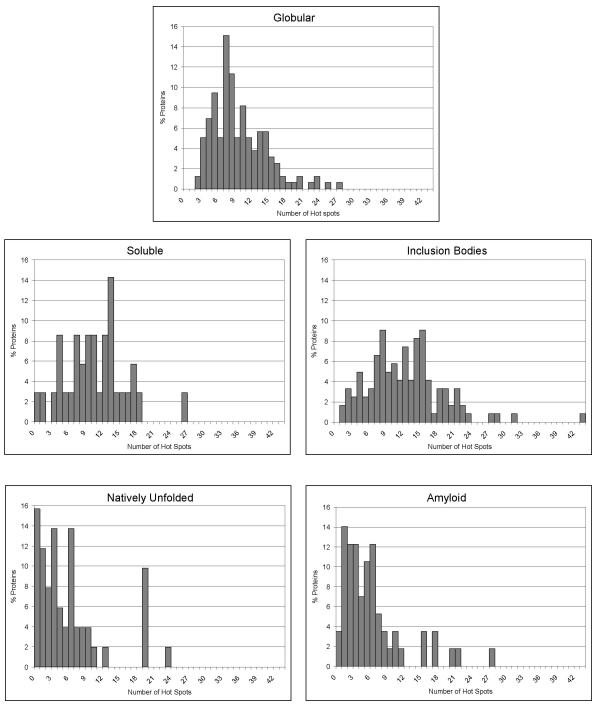
**"Hot spots" distribution in different protein groups**. Distribution of the number of "hot spots" relative to sequence length in the following protein datasets: natively globular proteins, intrinsically unstructured proteins, amyloidogenic proteins, soluble proteins when overexpressed in bacteria and proteins forming inclusion bodies when overexpressed in bacteria.

**Figure 5:**
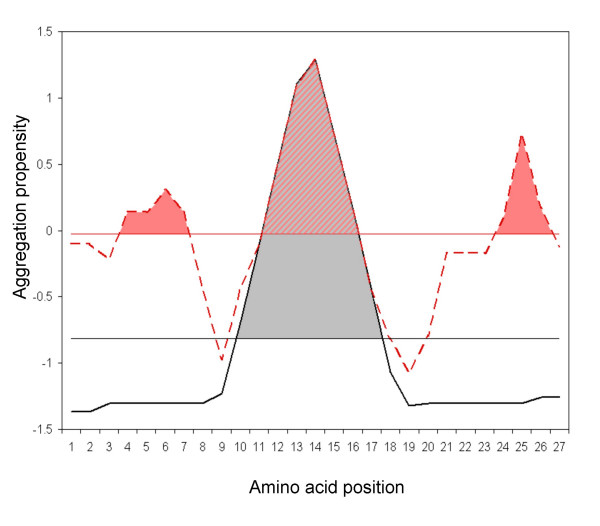
**Modulation of hot spot nucleation specificity by global aggregation propensity**. The black solid line represents a standard amyloidogenic protein aggregation profile, with only one "hot spot" and low global aggregation propensity. The pink discontinuous line corresponds to a typical aggregation profile from an inclusion-body-forming protein, with many "hot spots" and high global aggregation propensity. The horizontal lines represent the aggregation-propensity average thresholds for each sequence. The coloured regions indicate the area of each "hot spot" over the aggregation propensity threshold. It is proposed that a higher area over the threshold promotes a more specific aggregation reaction, resulting in highly ordered deposits.

It has been recently shown that proteins that form inclusion bodies (IB) upon recombinant overexpression in *E. coli *and proteins that form amyloids *in vivo *and/or *in vitro *share a good number of structural and functional features, including high purity of the aggregates, enrichment in beta-sheet structure, amyloid-tropic dye binding or enhanced proteolytic resistance [3]. Comparison of the two protein sets in search for similar trends in the predictions showed that, unexpectedly, the AGGRESCAN values for amyloid forming proteins are closer to those for IUP than for any other of the analysed datasets. Amyloid proteins have a lower Na^4^vSS and less HS than proteins in the IB or globular SCOP dataset (Figure 4). In contrast, the HSs in amyloid proteins comprise an area similar to those in IB or globular proteins, which is, however, significantly higher than the average HS area in IUP. These results suggests that, globally, the sequences of amyloidogenic proteins, like those of IUPs, have a low aggregation propensity, although the existence of specific aggregation-prone regions, absent or minor in IUPs, in a context in which they can act as specific and obligatory nucleation points from which the fibrillar structure could be expanded, finally results in highly ordered aggregates (Figure 4). This would explain why point mutations in the HSs of amyloidogenic proteins have usually a huge impact in their solubility, as they would modify the properties of one of the few points in the sequence that can promote and/or modulate aggregation. In contrast, the paradoxically higher-ranking global aggregation propensity of IB protein sequences is likely to indicate that here HS would play a less important role, since aggregation can also occur non specifically from many regions in the protein sequence. This would result in less structured deposits, and would also explain the rather moderate role of point mutations in IB aggregate formation. In other words, a given HS would promote specific amyloid formation in a low aggregating background, as its aggregation tendency outstands from the rest of the sequence. Conversely, the same HS needs to compete with the rest of the sequence to nucleate aggregation in a highly aggregating context (Figure 5). For the same reason unstructured aggregation is usually a much faster event than amyloidogenesis. Recent theoretical and experimental data support this view by showing that prevention of aggregation does not necessarily mean that amyloid fibril formation is abolished and *vice versa *[48]. This indicates that, despite the fact that aggregates and amyloid fibrils share many features, and the protein regions involved in their formation presumably intersect, they probably differ in the number and specificity of intermolecular contacts involved in the nucleation and stabilization of both types of polypeptide associations.

Recombinant protein production is an essential tool for the biotechnological industry and supports expanding areas of basic and biomedical research, including structural genomics and proteomics. The solubility of proteins expressed in bacteria under mass-production conditions is of major concern, since many recombinant polypeptides produced in bacteria accumulate as insoluble, often refractile, aggregates known as inclusion bodies (IBs) [49], excluding many biotechnologically relevant protein species from the market due to economically inconvenient yields. To date, the solubility of a given gene product has not been anticipated before gene expression. The comparison between the AGGRESCAN output values for proteins shown to be soluble under overexpression conditions in *E. coli *and those forming inclusion bodies shows that they share a similar number of HSs per 100 residues (NnHS), an expected output when considering that most proteins in both datasets are globular. However, IB-forming proteins have higher Na^4^vSS values than soluble proteins, suggesting that soluble proteins have, on average, a lower intrinsic aggregation tendency than IB-forming species, which may determine, at least partially, their relative behaviour upon overexpression. Overall, the predicted aggregation of proteins in the SCOP database is intermediate between that of soluble and insoluble proteins, suggesting that, in agreement with experimental observations, only a part of them would remain in the soluble cell fraction upon recombinant production. Although AGGRESCAN is able to catch the average trends in the aggregation of IBs and soluble protein groups, the individual outputs for proteins from both groups overlap significantly, making the prediction of the recombinant behaviour of a given sequence difficult in its present form. Besides, aggregation during recombinant production is the net result of several extrinsic and intrinsic factors, their relative importance depending on the protein and expression contexts.

## Conclusion

The software and web interface developed in the present study allow an easy and accurate identification and ranking of aggregation-prone regions in polypeptides. AGGRESCAN is also able to anticipate the effect of genetic or artificially introduced sequence changes on the aggregation properties of polypeptides. In addition to the investigation of the role of the primary sequence on protein aggregation and protein solubility, the algorithm can be used in the design of strategies for the treatment of amyloidogenesis, by targeting therapies to those regions in the polypeptide chain whose aggregation propensities outstand from the rest, provided that they are or become exposed to solvent in the disease-related protein conformation. The surprising observation that the aggregation propensities of amyloid sequences tend to be low, suggests that blocking the "hot spots" of aggregation in these proteins, either chemically or by mutation, may have a huge impact on their solubility. Interestingly enough, protein-protein interactions are often mediated through an energetic hot spot [50] which comprises few interface residues that contribute to most of the binding energy; identification and blocking of those sequence stretches has been suggested as an strategy to modulate protein interactions [51]. The ability of AGGRESCAN to analyze simultaneously the aggregation properties of large sets of protein sequences might be important for protein production in large-scale structural initiatives, for the analysis of the distribution of aggregation-prone regions in complete genomes or for evolutive studies, since it is likely that natural protein sequences have evolved in part to code for avoidance of aggregation.

## Availability and requirements

**Project name**: AGGRESCAN

**Project home page**: 

**Operating system(s)**: Platform independent

**Programming language**: a computing core coded in C and a front end written in a combination of html and perl cgi.

**Other requirements**: a web browser, such as Internet Explorer, Safari, or Firefox.

**Any restrictions to use by non-academics**: Incorporation into commercial products restricted.

## Authors' contributions

OCS implemented the software, NSG analyzed and prepared the final data and figures. FXA and JV contributed to data interpretation and manuscript redaction. XD directed the implementation of the software and contributed to manuscript redaction. SV directed the work and prepared the manuscript. All authors read and approved the final manuscript.

## Supplementary Material

Additional file 1 AGGRESCAN aggregation propensities Click here for file

Additional file 2 Help file of AGGRESCAN Click here for file

Additional file 3 Example of an output of AGGRESCAN Click here for file

Additional file 4 Protein data sets tested with AGGRESCAN Click here for file
