# Supplementary material for: AGGRESCAN: a server for the prediction and evaluation of "hot spots" of aggregation in polypeptides
Source: BMC Bioinformatics. 2007 Feb 27;8:65. doi: 10.1186/1471-2105-8-65 (PMC1828741; doi:10.1186/1471-2105-8-65)
Supplement: Additional file 1 — AGGRESCAN aggregation propensities [file 1471-2105-8-65-S1.pdf]

Additional File 1. Relative experimental aggregation propensities of the 20 natural amino acids derived from the analysis of the intracellular aggregation of mutants in the central position of the Central Hydrophobic Cluster in Amyloid-β-protein.

Amino acid	Aggregation propensities
I	1.822
F	1.754
V	1.594
L	1.38
Y	1.159
W	1.037
M	0.91
C	0.604
A	-0.036
T	-0.159
S	-0.294
P	-0.334
G	-0.535
K	-0.931
H	-1.033
Q	-1.231
R	-1.24
N	-1.302
E	-1.412
D	-1.836
