# Supplementary material for: AGGRESCAN: a server for the prediction and evaluation of "hot spots" of aggregation in polypeptides
Source: BMC Bioinformatics. 2007 Feb 27;8:65. doi: 10.1186/1471-2105-8-65 (PMC1828741; doi:10.1186/1471-2105-8-65)
Supplement: Additional file 2 — Help file of AGGRESCAN [file 1471-2105-8-65-S2.pdf]

Additional file 2

Help file of AGGRESCAN

Sequence Name

Name given by the user to the input amino-acid sequence.

Graphics

1) Profile graphic : a^4v (red) , a^3vSA (green line) and HST (blue) as function of amino acid sequence.

2) Area Graphic HSA as function of amino acid sequence. If there are no hot spots, the graphic is displayed.

3) Normalized-Area graphic NHSA for 100 residues as function of amino acid sequence.

amino-acid aggregation-propensity value. (a^3v)

N. Sánchez de Groot et al. Prediction of "hot spots" of aggregation in disease-linked polypeptides. BMC Structural Biology 2005, 5:18. See table for actual values.

a^3v Sequence Average. (a^3vSA)

Average of the a^3v over the entire input amino-acid sequence.

Hot Spot Threshold (HST)

Average of the result of multiplying the a^3v of each of the 20 natural amino acids by its frequency in the SwissProt database. Its current value is -0.02.

a^3v window average (a^4v)

a^3v average over a sliding window of 5, 7, 9 or 11 residues (depending on total sequence length). The resulting value is assigned to the central residue in the window. In this way, all but the 2, 3, 4 or 5 N- and C-terminal residues (for a 5-, 7-, 9- or 11-residue window, respectively) are assigned an a4v. The 2 extreme residues are given specific a4v to account for charge effects. The remaining off-centre N- and C-terminal residues are assigned the a4v calculated for the first and last window centres, respectively.

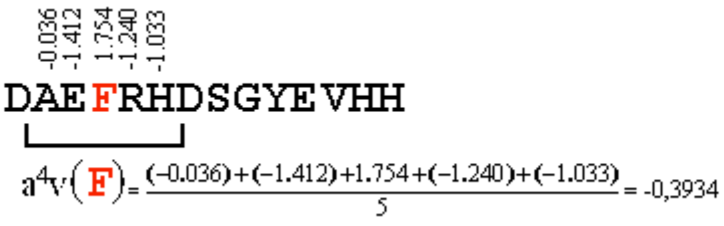

Agregation Profile (AP)

Sequence of the a^4v for the input amino-acid sequence.

Hot Spot (HS)

Region with 5 or more residues on sequence with an a^4v larger than HST and no proline (aggregation breaker).

Number of Hot Spots (nHS)

Number of HS in the input amino-acid sequence.

Normalized number of Hot Spots for 100 residues (NnHS)

nHS divided by the number of residues in the input sequence and multipled by 100

a^4v average in the Hot Spot (a^4vAHS)

a^4v average in a given HS.

Total Area of the aggregation profile (TA)

Area of the AP (taking HST as the zero axis) along the entire input amino-acid sequence, calculated with trapezoidal integration (midpoint rule).

Area of the Aggregation Profile above the hot-spot Threshold (AAT)

Area of the AP above the HST along the entire input amino-acid sequence, calculated with trapezoidal integration(midpoint rule).

AAT per residue (AATr)

AAT divided by the number of residues in the input amino-acid sequence.

Hot-Spot Area (HSA)

Area of the AP above the HST in a given HS, calculated with trapezoidal integration (midpoint rule). The HSA of a residue in a hot spot is set equal to the HSA of the hot spot.

Normalized Hot-Spot Area per residue (NHSA)

HSA divided by the number of residues in the input sequence.

Total Hot-Spot Area (THSA)

Sum of the HSA of the individual HS.

THSA per residue (THSAr)

THSA divided by the number of residues in the input amino-acid sequence.

AA

This column is the amino acid sequence of the protein

a^4v Sequence Sum (a^4vSS)

Sum of a^4v over the entire input amino-acid sequence.

Normalized a4vSS for 100 residues (Na4vSS)

a4vSS divided by the number of residues in the input amino-acid sequence and multiplied by 100.
