# Supplementary material for: AGGRESCAN: a server for the prediction and evaluation of "hot spots" of aggregation in polypeptides
Source: BMC Bioinformatics. 2007 Feb 27;8:65. doi: 10.1186/1471-2105-8-65 (PMC1828741; doi:10.1186/1471-2105-8-65)
Supplement: Additional file 3 — Example of an output of AGGRESCAN [file 1471-2105-8-65-S3.pdf]

Additional file 3

Example of an output of AGGRESCAN

Output Example

Sequence Name:	sh3	sh3n47a	Average over all sequences
Graphics:			
a3v Sequence Average (a3vSA):	-0.126	-0.105	-0.115
Number of Hot Spots (nHS):	3	4	3.500
Normalized nHS for 100 residues (NnHS):	4.839	6.452	5.645
Area of the profile Above Threshold (AAT):	11.068	11.250	11.159
Total Hot Spot Area (THSA):	9.183	10.247	9.715
Total Area (TA):	-6.674	-5.408	-6.041
AAT per residue (AATr):	0.179	0.181	0.180
THSA per residue (THSAr):	0.148	0.165	0.157
Normalized a4v Sequence Sum for 100 residues (Na4vSS):	-13.9	-11.9	-12.900

sh3

#	AA	a4v	HSA	NHSA	a4vAHS
1	m	-0.824	0.000	0.000	0.000
2	d	-0.824	0.000	0.000	0.000
3	e	-0.606	0.000	0.000	0.000
4	t	-0.975	0.000	0.000	0.000
5	g	-0.890	0.000	0.000	0.000
6	k	-0.331	0.000	0.000	0.000
7	e	0.019	4.298	0.537	0.517
8	l	0.402	4.298	0.537	0.517
9	v	0.581	4.298	0.537	0.517
10	l	1.140	4.298	0.537	0.517
11	a	1.095	4.298	0.537	0.517
12	l	0.409	4.298	0.537	0.517
13	y	0.365	4.298	0.537	0.517
14	d	0.126	4.298	0.537	0.517
15	y	-0.432	0.000	0.000	0.000
16	q	-0.850	0.000	0.000	0.000
17	e	-0.542	0.000	0.000	0.000
18	k	-0.840	0.000	0.000	0.000
19	s	-0.842	0.000	0.000	0.000
20	p	-0.842	0.000	0.000	0.000
21	r	-0.337	0.000	0.000	0.000
22	e	-0.310	0.000	0.000	0.000
23	v	-0.061	0.000	0.000	0.000
24	t	0.000	0.137	0.000	0.049
25	m	0.097	0.137	0.000	0.049
26	k	-0.329	0.000	0.000	0.000
27	k	-0.665	0.000	0.000	0.000
28	g	-0.482	0.000	0.000	0.000
29	d	-0.020	0.000	0.000	0.000
30	i	0.134	2.870	0.478	0.458
31	l	0.517	2.870	0.478	0.458
32	t	1.161	2.870	0.478	0.458
33	l	0.536	2.870	0.478	0.458
34	l	0.201	2.870	0.478	0.458
35	n	0.201	2.870	0.478	0.458
36	s	-0.335	0.000	0.000	0.000
37	t	-0.798	0.000	0.000	0.000
38	n	-0.904	0.000	0.000	0.000
39	k	-0.638	0.000	0.000	0.000
40	d	-0.399	0.000	0.000	0.000
41	w	-0.325	0.000	0.000	0.000
42	w	0.180	0.882	0.000	0.274
43	k	0.265	0.882	0.000	0.274
44	v	0.376	0.882	0.000	0.274
45	e	-0.091	0.000	0.000	0.000
46	v	-0.272	0.000	0.000	0.000
47	N	-0.839	0.000	0.000	0.000
48	d	-0.803	0.000	0.000	0.000
49	r	-1.229	0.000	0.000	0.000
50	q	-0.618	0.000	0.000	0.000
51	g	0.068	0.867	0.000	0.269
52	f	0.250	0.867	0.000	0.269
53	v	0.489	0.867	0.000	0.269
54	p	0.588	0.000	0.000	0.000
55	a	0.469	2.014	0.403	0.383
56	a	0.469	2.014	0.403	0.383
57	y	0.350	2.014	0.403	0.383
58	v	0.171	2.014	0.403	0.383
59	k	0.454	2.014	0.403	0.383
60	k	-0.145	0.000	0.000	0.000
61	l	-0.681	0.000	0.000	0.000
62	d	-0.681	0.000	0.000	0.000

sh3n47a

#	AA	a4v	HSA	NHSA	a4vAHS
1	m	-0.824	0.000	0.000	0.000
2	d	-0.824	0.000	0.000	0.000
3	e	-0.606	0.000	0.000	0.000
4	t	-0.975	0.000	0.000	0.000
5	g	-0.890	0.000	0.000	0.000
6	k	-0.331	0.000	0.000	0.000
7	e	0.019	4.298	0.537	0.517
8	l	0.402	4.298	0.537	0.517
9	v	0.581	4.298	0.537	0.517
10	l	1.140	4.298	0.537	0.517
11	a	1.095	4.298	0.537	0.517
12	l	0.409	4.298	0.537	0.517
13	y	0.365	4.298	0.537	0.517
14	d	0.126	4.298	0.537	0.517
15	y	-0.432	0.000	0.000	0.000
16	q	-0.850	0.000	0.000	0.000
17	e	-0.542	0.000	0.000	0.000
18	k	-0.840	0.000	0.000	0.000
19	s	-0.842	0.000	0.000	0.000
20	p	-0.842	0.000	0.000	0.000
21	r	-0.337	0.000	0.000	0.000
22	e	-0.310	0.000	0.000	0.000
23	v	-0.061	0.000	0.000	0.000
24	t	0.000	0.137	0.000	0.049
25	m	0.097	0.137	0.000	0.049
26	k	-0.329	0.000	0.000	0.000
27	k	-0.665	0.000	0.000	0.000
28	g	-0.482	0.000	0.000	0.000
29	d	-0.020	0.000	0.000	0.000
30	i	0.134	2.870	0.478	0.458
31	l	0.517	2.870	0.478	0.458
32	t	1.161	2.870	0.478	0.458
33	l	0.536	2.870	0.478	0.458
34	l	0.201	2.870	0.478	0.458
35	n	0.201	2.870	0.478	0.458
36	s	-0.335	0.000	0.000	0.000
37	t	-0.798	0.000	0.000	0.000
38	n	-0.904	0.000	0.000	0.000
39	k	-0.638	0.000	0.000	0.000
40	d	-0.399	0.000	0.000	0.000
41	w	-0.325	0.000	0.000	0.000
42	w	0.180	1.064	0.213	0.193
43	k	0.265	1.064	0.213	0.193
44	v	0.376	1.064	0.213	0.193
45	e	0.162	1.064	0.213	0.193
46	v	-0.019	1.064	0.213	0.193
47	A	-0.586	0.000	0.000	0.000
48	d	-0.550	0.000	0.000	0.000
49	r	-0.976	0.000	0.000	0.000
50	q	-0.618	0.000	0.000	0.000
51	g	0.068	0.867	0.000	0.269
52	f	0.250	0.867	0.000	0.269
53	v	0.489	0.867	0.000	0.269
54	p	0.588	0.000	0.000	0.000
55	a	0.469	2.014	0.403	0.383
56	a	0.469	2.014	0.403	0.383
57	y	0.350	2.014	0.403	0.383
58	v	0.171	2.014	0.403	0.383
59	k	0.454	2.014	0.403	0.383
60	k	-0.145	0.000	0.000	0.000
61	l	-0.681	0.000	0.000	0.000
62	d	-0.681	0.000	0.000	0.000

Sorted by Na4vSS

sh3n47a	-11.90
sh3	-13.90
