# Supplementary material for: AGGRESCAN: a server for the prediction and evaluation of "hot spots" of aggregation in polypeptides
Source: BMC Bioinformatics. 2007 Feb 27;8:65. doi: 10.1186/1471-2105-8-65 (PMC1828741; doi:10.1186/1471-2105-8-65)
Supplement: Additional file 4 — Protein data sets tested with AGGRESCAN [file 1471-2105-8-65-S4.pdf]

Additional file 4 Protein data sets tested with AGGRESCAN

Globular proteins (SCOP) data set[a]

>d1dlwa 1	>d1kcw1	>d1ri6a	>d1fxxa	>d1ehya	>d1i50a	>d1oc7a	>d2nmba
>d1s69a 1	>d1sddb2	>d1olza2	>d1j9aa	>d1llfa	>d1k8ta	>d1m65a	>d1q67a
>d1idra	>d1ikop	>d1shyb1	>d1b8oa	>d1qj4a	>d1vdda	>d1l6ra	>d1es9a
>d1ngka	>d1so9a	>d1g72a	>d1q1ga	>d1kica	>d1kq3a	>d1uj6a1	>d1h05a
>d1kr7a	>d2viua	>d1dosa	>d1jwqa	>d1gy7a	>d1slqa	>d1soua	>d1k7ka
>d1bgf 90	>d1szia	>d1ohla	>d1di6a	>d1nwwa	>d1acc	>d1oxwa	>d2dpma
>d1ui5a1	>d1ug7a	>d1of8a	>d1m72a	>d1ktua	>d1vjua	>d1h1da	>d1inla
>d1t33a1	>d1uw4b	>d1kkoa1	>d1nw9b	>d1lqva	>d1lm5a	>d1nt2a	>d1th0a
>d1rr7a	>d1sdia 198	>d1jqna	>d1vhua	>d1s1qa	>d1sw6a	>d1qyra	>d1szaa
>d1mtyg 233	>d1r75a	>d1l1la	>d1xvaa c665	>d1gx1a	>d1lkxa	>d1k75a	>d1q2za
>d1om2a 234	>d1pm4a	>d1r61a	>d1jqea c669	>d1q8ra	>d1ku1a	>d1jixa	>d1itha
>d1jw2a 235	>d1aol	>d1kid	>d1l3ia c6622	>d1dwna d85	>d1klxa	>d1rrea	>d1q1fa
>d1m1eb	>d1utya	>d1gmla	>d1nw3a	>d1l5ia	>d1ld8a	>d1vd2a	>d1e31a
>d1gs9a	>d1aly	>d1vjza	>d1jg1a	>d1omha	>d1qjba	>d1m2xa	>d1jtia
>d1jkva 253	>d1fl0a	>d1osna	>d1m32a	>d1utea	>d1hh8a	>d1utea	>d1tlja
>d1rtyb	>d1d7qa	>d1zin1	>d2gsaa	>d1auia	>d1n08a	>d1t7ra	>d1p0ha
>d1noga	>d1gpc	>d1vhta	>d2oata	>d1uf5a	>d1d1na	>d1ie9a	>d1cjwa
>d1niga	>d1e9ga	>d1kaga	>d1m7ya	>d1i7qa	>d1jj2b	>d1ak0	>d1bo4a
>d1bgc	>d1k8qa	>d1m7ga	>d1ga8a	>d1ae9a	>d1k0ha	>d1shwa	>d1fw9a
>d1gw0a3	>d1jofa	>d1musa	>d1wm1a	>d1u09a e84	>d1ewna	>d1t2sa	>d1puja

Inclusion bodies forming proteins data set[b]

>1xat	>p9752	>P28784	>CAA84850	>AAB64992	> CAA90962	>atCKA1
>AAA03018	>q97q4	>Q06700	>CAA44325	>AAC35427	> 2107187A	
>AAC36914	>s50990	>P56007	>CAC42773	>BAA08203	> AAB07708	
>AAD15038	>CAA49762	>P56006	>CAB43519	>BAA31582	>AAA93168	
>AAG18010	>CAC11023	>P37698	>CAB43097	>2TMP	> AAA66345	
>AAO83646	>CAC11021	>P43387	>Q27607	>1LQH	>I51190	
>AAP74699	>CAA74917	>P37747	>NRASMR	>1DYR	>NP_031475	
>BAB37623	>CAA48684	>P09169	>JC7310	> AAC60368	>NP_005567	
>BAC21259	>CAA00187	>P02933	>JC4990	> AAC53663	>NP_005428	
>CAA00187	>P04575	>P82604	>JC6005	> AAC52514	>2PRN	
>caa31649	>P02643	>Q47278	>S32494	> AAC43487	>AAP47198	
>caa94639	>Q9ULZ9	>AAB25034	>A42983	> AAC48984	>AAA98747	
>cac80860	>Q9ESI9	>P36955	>CAC42505	> AAC38578	>AAL07473	
>np_00622	>P49275	>P55916	>CAA38080	> AAC34292	>AAZ85393	
>p02643	>P97521	>P55851	>CAA41526	>CAA73028	>NP_001011	
>p04575	>P13271	>P39767	>P07713	> CAB06820	>ntCKA3	
>p18417	>NP_006221	>CAA63058	>AAL96679	> 1SIP	>ntCKA2	
>p21685	>NP_005944	>CAA98669	>AAD09856	>BAA61829	>ntCKA1	
>p21867	>P33303	>CAA96855	>CAA11075	> AAB71336	>ntCKB1	
>p82604	>O68983	>CAA90967	>AAF63204	>BAA02440	>atCKA2	

Soluble proteins data set[b]

>1fsz	>O31616
>AAA99492	>CAA06715
>AAB66502	>P22315
>AAM14744	>1AF3
>BAA92530	>2TPT
>BAB03311	>AAA66289
>CAA82319	>AAC45645
>CAD42331	>BAB68505
>NP_217055	>NP_438572
>NP_217744	>P31853
>NP_559383	>Q42539
>P08980	>n-terminalntCKB1
>P22061	>NP_417795
>P51570	>CAC20129
>P60716	>AAN81585
>NP_201242	>NP_003320
>NP_312918	>AAB53783
>P04789	>CAA00084
>P15999	
>P09743	

Unstructured proteins data set[b]

>NP_000537	>Q13541	>P16439T(*)
>NP_000086	>P09803	>P71413(*)
>NP_001759	>P04571	>P69346
>NP_002814	>P46527	>P32499
>NP_009292	>P19429	>NP_004210
>NP_031695	>H64966	>P24588
>NP_013439	>S48708	>CAA66701
>NP_006749	>AAM10853	>AAB08709
>NP_000336	>CAB59720	>NP_736592
>NP_005293	>AAH00312	>AAB50256
>P40316	>BAD20777	>Q27957
>NP_895627	>AAQ20894	
>AAB27444	>CAA65311	
>AAB33495	>AAL18843	
>P11137	>1A87	
>P70445	>AAC45264	
>P03045	>AAC02114	
>P00560	>AAB08709	
>Q03768	>CAA42717	
>P43532	>CAA42650	

Amyloid proteins data set[b]

>spectrin	>P51446	>P10997
>NP_000537	>P40252	>AAQ67734
>NP_009292	>P06396	>AAC33174
>NP_004039	>NP_000474	>1AG2
>NP_000336	>NP_776351	>1LOZ
>NP_000475	>NP_062042	>BAA00011
>P_002814	>NP_036718	>CAA67766
>P02766	>NP_035448	>AAB41502
>YLDGA	>P10636	>1GXUA
>P05367	>AAH655291	>BAA05124
>AAK26231	>P01034	>1MVK_G
>3PTE	>AAB28602	>Q9Y287
>1GGTB	>AAB20537	>NP_612457
>1SPF	>P04156	>Q08431
>2EQL	>A609521	>NP_002334
>1TCA	>A23843	>AAB97519
>1B10(*)	>S05037	>Q7SIF8
>CAD91183	>AAH20795	
>NP_031497	>AAH192121	
>P14621	>P19707	

Protein code:
(a) SCOP dataset identifies (*sid* format)
(b) NCBI dataset accession number

(*) This record has been discontinued.
